# Sol-gel Syntheses of Photocatalysts for the Removal of Pharmaceutical Products in Water

**DOI:** 10.3390/nano9010126

**Published:** 2019-01-20

**Authors:** Artium Belet, Cédric Wolfs, Julien G. Mahy, Dirk Poelman, Christelle Vreuls, Nathalie Gillard, Stéphanie D. Lambert

**Affiliations:** 1 Department of Chemical Engineering–Nanomaterials, Catalysis, Electrochemistry, University of Liege, Building B6a, B-4000 Liege, Belgium; Artium.Belet@uliege.be (A.B.); cedric.wolfs@uliege.be (C.W.); Stephanie.lambert@uliege.be (S.D.L.); 2 Lumilab, Department of Solid State Sciences, Ghent University, Krijgslaan 281-building S1, 9000 Ghent, Belgium; Dirk.Poelman@UGent.be; 3 Celabor, Research Centre, Environmental Department, B-4650 Herve, Belgium; Christelle.Vreuls@celabor.be; 4 CER Groupe, Health Department, B-6900 Marloie, Belgium; n.gillard@cergroupe.be

**Keywords:** TiO_2_, photocatalysis, sol-gel process, thin films, pharmaceutical products

## Abstract

A screening study on seven photocatalysts was performed to identify the best candidate for pharmaceutical products degradation in water. Photocatalysts were deposited as thin films through a sol-gel process and subsequent dip-coating on glass slides. The efficiency of each photocatalyst was assessed through the degradation of methylene blue first, and then, through the degradation of 15 different pharmaceutical products. Two main types of synthesis methods were considered: aqueous syntheses, where the reaction takes place in water, and organic syntheses, where reactions take place in an organic solvent and only a stoichiometric amount of water is added to the reaction medium. Photocatalysts synthesized via aqueous sol-gel routes showed relatively lower degradation efficiencies; however, the organic route required a calcination step at high temperature to form the photoactive crystalline phase, while the aqueous route did not. The best performances for the degradation of pharmaceuticals arose when Evonik P25 and silver nanoparticles were added to TiO_2_, which was synthesized using an organic solvent. In the case of methylene blue degradation, TiO_2_ modified with Evonik P25 and TiO_2_ doped with MnO_2_ nanoparticles were the two best candidates.

## Highlights

- Aqueous and organic synthesis pathways by sol-gel process for photocatalysts.
- Photodegradation of organic molecules in aqueous media.
- Ag NP-doped TiO_2_ (anatase) promising for the degradation of pharmaceutical compounds.

## 1. Introduction

In recent years, concerns about hazards of pharmaceutical compounds found in treated wastewaters, surface water, ground water and drinking water have risen. The impact of such pollutants in the environment has been extensively studied in the literature and has been acknowledged to constitute a health risk for humans and ecosystems. The major threats result from a long-term exposure of non-target organisms in contact with those pharmaceutically-active agents. Among them, endocrine-disruptive chemicals (EDCs) induce failures of the endocrine system in living organisms, leading to perturbations of their growth, physiology, and reproductive tract [1,2,3,4]. Another disastrous effect of the continual exposure to EDCs is the high spread of multidrug-resistant bacteria, especially antibiotics-resistant bacteria [5,6,7,8].

As a consequence of the anthropogenic activities in urban households and clinical environments, effluents coming from wastewater treatment plants (WWTPs) are polluted with pharmaceuticals in a range from ng/L to µg/L. In fact, it is now well-established that traditional primary, secondary and tertiary physical, chemical and biological treatments in a WWTP do not allow to completely remove complex organic compounds that end up in effluents and sludges. Therefore, there is a real need for an additional efficient treatment process to degrade these pharmaceuticals before they reach the ecosystem.

With the aim to degrade pharmaceutical compounds in WWTP effluents, the development of so-called quaternary treatments is today very important. Among these quaternary treatments, in which ozone injection (O_3_) and UV illumination can be coupled, Advanced Oxidation Processes (AOP) are cited [9,10,11]. It is also possible to use heterogeneous photocatalysts such as TiO_2_ and ZnO to increase the efficiency of the purification processes [9,10,11]; in this case, a highly specific surface area is usually needed [12].

The objective of the study was to identify the most efficient sol-gel produced thin film heterogeneous photocatalyst among seven materials, using exclusively inexpensive materials and methods.

Four criteria were identified for that purpose: (i) the sol-gel method had to be used because it is a soft chemistry synthesis method (room temperature and pressure); (ii) the photocatalyst was active under UV light; (iii) the photocatalyst could be deposited as a thin layer onto glass; (iv) the price of the synthesis had to be relatively inexpensive. In this way, the goal of this paper is to rank the most promising photocatalysts for a scaled-up application, such as a wastewater treatment plant. Thus, the fundamental nature of the solvents and dopants used did not matter as much as their availability, price, and easiness of handling.

While the objectives (i) and (iv) are related to the cost of the method, (ii) and (iii) are related to the prospect of industrialization. Indeed, objective (ii) corresponds to the most usual type of light chosen for wastewater treatment plants using AOP. Objective (iii) is necessary to make sure the photocatalyst is recoverable.

Photocatalysts are often semiconductors, which means that by a sufficient external energy supply, an electron is promoted from the valence band (VB) to the conduction band (CB). Overcoming the band-gap energetic barrier or “band-gap” (BG), the electron leaves a positive hole in the VB and forms what is commonly called an “electron-hole pair” (e^−^-h^+^ pair (1)) [9,10].

(1) $$\mathrm{Photocatalyst}+h\nu_{\mathrm{MIN}}\to e_{\mathrm{CB}}^{-}+h_{\mathrm{VB}}^{+}$$

where h is the Planck constant (h = 6.626 × 10^−34^ ·) and ν is the frequency (Hz) of the incident light. For example, the amount of energy required to activate the TiO_2_ is high with respect to the width of the band gap (3.2 eV, corresponding to a wavelength of 388 nm, for the anatase crystalline phase) and corresponds to the UV light. When the photo-generated e^−^-h^+^ pairs are at the surface of the photocatalyst, they produce radical species, •O_2_^−^ (superoxides) and •OH, through reactions with adsorbed O_2_ and H_2_O (2) and (3). Those radicals react with the pollutant and induce its degradation (4) [9,13,14].

(2) $$2\;H_{2}O+\;h_{\mathrm{VB}}^{+}\to \;•\mathrm{OH}+H_{3}O^{+}$$

(3) $$O_{2}+e_{\mathrm{CB}}^{-}\to \;•O_{2}^{-}$$

(4) $$\mathrm{Pollutant}+h_{\mathrm{VB}}^{+}\to {\mathrm{Pollutant}}_{\mathrm{OX}}$$

However, the high degree of recombination between photogenerated electrons and holes is a major rate-limiting factor controlling the photocatalytic efficiency. For instance, the characteristic time for “e^−^-h^+^ pair” recombination in TiO_2_ ranges from 10 ns to 100 ns, while it is in the range of 1 fs for “e^−^-h^+^ pair” generation [13]. Attempts to increase the TiO_2_ efficiency were made by doping with transition metals such as Pd, Au and Ag [15,16]. Those dopants act as electron-traps, thus increasing the lifetime of electrons available for radical formation reactions. Some additives also modify the surface properties of the photocatalyst which could increase the pollutants adsorption and favor the interfacial reactions [11,15,16,17]. It is also worth mentioning that both the band gap and the photocatalytic efficiency can be affected by the dispersion of the catalyst on its support and by the particle size of the photocatalyst [18].

For several years, the sol-gel process has been used for the synthesis of TiO_2_ or ZnO in the form of powders or films which allows for the control of the nanostructure and surface properties [17,18,19,20]. In order to obtain TiO_2_ by a sol-gel process, two paths can be considered depending on the solvent used: a non-aqueous solvent and an aqueous solvent.

In a non-aqueous solvent, the sol-gel synthesis is conducted in an organic solvent able to complex the titania precursor (Ti-(OR)_4_) to control its reactivity. A stoichiometric amount of water is added to avoid precipitation [20]. Then, the material undergoes a drying and calcination step to remove residual organic molecules and to crystallize amorphous TiO_2_ in anatase or rutile crystalline phases, depending on the processing conditions and temperature.

In an aqueous process, water is the solvent and is present in large excess. In this synthesis, peptizing agents are used to form small TiO_2_-anatase nanoparticles at ambient temperature [14,19]. The main advantages of this route are the use of water as a solvent and that an anatase phase was obtained at low temperature. This method is thus well suited for industrialization.

Fifteen pharmaceuticals were selected based on the European Union directive 2008/105/CE “on environmental quality standards in the field of water policy” [21], and also according to their low biodegradability, a higher solubility in water than in sludge and a large occurrence in most common WWTP effluents.

Photocatalysts were deposited by dip-coating on alkali-free glass slides as coatings by either aqueous or organic sol-gel processes. Catalytic performances were investigated on zinc oxide (ZnO), titanium dioxide (TiO_2_) and modified titanium dioxide (by addition of Ag, Evonik P25, and MnO_2_-nanoparticles and Zn^2+^ ions).

Coatings were characterized by profilometry for thickness and roughness by grazing incidence X-ray diffraction (GIXRD) for photoactive crystalline phase presence, by diffuse reflectance from corresponding powders for band-gap determination, by ICP-AES for quantitative analysis of dopants, and by a methylene blue degradation test as a photoactivity efficiency benchmark.

## 2. Experimental

### 2.1. Material Preparation

The following reagents were used for both aqueous and organic sol-gel syntheses:

Titanium(IV) tetraisopropoxide (TTiP, Sigma-Aldrich, ≥97.0%, St. Louis, MO, USA), acetic acid glacial (aldehyde free) (HAc, Fisher Scientific, Hampton, NH, USA), nitric acid 65% (HNO_3_, Merck, Suprapur, Fort kennerworth, NJ, USA), reagent-grade milliQ water (m-H_2_O, purified by a Purelab Flex device from Veolia Water, 18.2 MΩcm, Paris, France) or deionized water (d-H_2_O), isopropanol (i-PrOH, Acros Organics, 99.5%, Extra dry over Molecular Sieves, Fisher Scientific), zinc nitrate hexahydrate (Zn(NO_3_)_2_∙6H_2_O, Sigma-Aldrich, crystallized ≥99.0% (KT)), 2-methoxyethanol (MetOH, Sigma-Aldrich, anhydrous 99.8%), silver acetate (AgAc, Aldrich Chemical Company, 99.999% light-sensitive), *N*-[3-(Trimethoxysilyl)propyl]ethylenediamine (EDAS, Sigma-Aldrich, 97%), commercial TiO_2_ Evonik P25 (P25, purity of 99%, 85% anatase and 15% rutile [22]), absolute ethanol (a-Eth, J.T. Baker Exceeds ACS Specifications, Fisher Scientific), zinc acetate dehydrate (Zn(CH_3_COO)_2_∙2H_2_O, Sigma-Aldrich, ACS reagent ≥98%) and monoethanolamine (MEA, Sigma-Aldrich, purified by redistillation ≥99.5%). Ramsdellite-MnO_2_ nanoparticles (MnO_2_ NP) were synthesized through a method described by Portehault et al. [23].

#### 2.1.1. Aqueous Sol-gel Synthesis

Pure titanium dioxide aqueous sol, called TiO_2_ aq., was synthesized through procedures adapted from Malengreaux et al. [24] and by Bartlett et al. [25]. First, HAc was added to TTiP (molar ratio 1:1) and the solution was gently stirred for 15 min at room temperature in a closed container. HAc, like most molecules that contain carboxylic acid functional groups, was used as a stabilizer against the rapid hydrolysis reactions occurring with titanium alkoxides [26,27,28]. The solution of HAc/TTiP was then added to a large excess of water (molar ratio m-H_2_O: TTiP of 110) under vigorous stirring, yielding a white precipitate. After 10 min. of decantation, the supernatant was removed and the same volume of water was added on the slurry under vigorous stirring. The washing cycle was performed two more times in order to remove the HAc, the isopropanol and the isopropyl acetate. Finally, the same volume of water with HNO_3_ (1 mol/L) was added and the solution was left under vigorous stirring at 45 °C for 16 h. HNO_3_ was used as a peptizing agent [14,29], allowing nanocrystallites of titania to be dispersed due to repulsive electrostatic charges on their surface. Hydrothermal and peptization conditions as well as the use of a stabilizer led to the formation of anatase titania nanocrystallites [30]. At the end, a blueish transparent sol was obtained and was kept in ambient atmosphere.

Zinc-doped titanium dioxide sol TiO_2_ + Zn^2+^ was synthesized according to a procedure described by Mahy et al. [14]. First, TTiP was dissolved in isopropanol (molar ratio i-PrOH: TTiP = 11) and stirred vigorously at room temperature for 30 min. Secondly, a solution of deionized water acidified by HNO_3_ to pH = 1 was prepared (molar ratio d-H_2_O: TTiP = 760). Then, the corresponding nitrate salt was added to the acidic solution (molar ratio Zn(NO_3_)_2_∙6H_2_O: TTiP = 0.005). This solution was poured into a larger container pre-heated at 80 °C using a silicone oil bath. Finally, the solution of TTiP/i-PrOH was rapidly added by a syringe and the mixture was kept at 75 °C under vigorous stirring for 12 h. After 12 h, a blueish transparent sol was obtained. It was kept in ambient atmosphere.

Sols made by aqueous procedures were used at room temperature in ambient atmosphere. Due to their high stability toward aggregation and polymerization thanks to the HNO_3_ peptization, they could be stored for several months without undergoing gelation.

#### 2.1.2. Organic Sol-gel Synthesis

Pure titanium dioxide sol TiO_2_ org. was synthesized according to Bodson et al. [17,20,31]. Molar quantities of reactants were obtained by solving hydrolysis and dilution rate equations, H = n_H2O_/n_TTiP_ and D = n_MetOH_/n_TTiP_, leading to H = 2.2 and D = 44 respectively. Two solutions, one of half the volume of MetOH and TTiP and the second with reagent-grade milliQ water were vigorously stirred at room temperature for 30 min. Both solutions were then mixed together and stirred for 30 min. After 30 min, the sol was perfectly transparent as it was synthesized under an inert N_2_ atmosphere. Both syntheses led to similar characteristics of films after dip-coating and calcination steps.

Silver nanoparticle-doped titanium dioxide sol TiO_2_ + Ag was prepared through a co-gelation modified procedure described by Braconnier et al. [16]. The following equations have been solved in order to obtain the molar quantities to be used:

(5) $$H=\frac{n_{m-H_{2}O}}{n_{\mathrm{TTiP}}+\frac{3}{4}n_{\mathrm{EDAS}}}=2.2$$

(6) $$D=\frac{n_{\mathrm{MetOH}}}{n_{\mathrm{TTiP}}+n_{\mathrm{EDAS}}}=40$$

(7) $${\%\mathrm{wt}}_{\mathrm{Ag}}=\frac{n_{\mathrm{Ag}}{\mathrm{MM}}_{\mathrm{Ag}}}{n_{\mathrm{Ag}}{\mathrm{MM}}_{\mathrm{Ag}}+n_{\mathrm{TTiP}}{\mathrm{MM}}_{{\mathrm{TiO}}_{2}}+n_{\mathrm{EDAS}}{\mathrm{MM}}_{{\mathrm{SiO}}_{2}}}=1\%$$

(8) $$\frac{n_{\mathrm{EDAS}}}{n_{\mathrm{Ag}}}=2$$

The molar ratio of EDAS over Ag was set to a value of 2 in order to be in an excess of ligand with respect to silver ions, ensuring a complete complexation reaction as explained by Lambert et al. [32]. The volume of solvent V_MetOH_ was set to 120 mL. Besides its properties of stabilizing the sol, MetOH was also used as a reducing agent for the silver ions [33,34]. First, a solution of silver acetate dispersed in half the volume of V_MetOH_ was stirred at room temperature for 30 min under N_2_ atmosphere. Subsequently, the complexing agent EDAS was added to the whitish solution and it was stirred for another 30 min. Finally, TTiP was added and the resulting mixture was stirred for another 30 min. In parallel, milliQ water m-H_2_O was added to the remaining volume of V_MetOH_ and stirred for 30 min before being added to the latter mixture. After 30 min of vigorous stirring, the yellowish transparent obtained sol was ready to be used.

Titanium dioxide modified with P25 or doped with MnO_2_ NP sols, respectively TiO_2_ + P25 and TiO_2_ + MnO_2_ NP, were synthesized as follows, with V_MetOH_ = 90 mL. Under N_2_ atmosphere, a solution of TTiP and half the volume of MetOH (molar ratio ½ MetOH: TTiP = 22) was stirred at room temperature for 30 min. Before P25 or MnO_2_ NP, m_P25_/m_TiO_2__ = 10% and m_MnO_2__/m_TiO_2__ = 5% respectively were added, and the resulting mixture was stirred for another 30 min. In parallel, a solution of milliQ water m-H_2_O and the remaining half of the volume of MetOH (molar ratio ½ MetOH: m-H_2_O = 10) was stirred for 30 min at room temperature. Both solutions were then mixed together and left under vigorous stirring for 16 h at 50 °C. After this time, the resulting sols were both completely opaque with a white (TiO_2_ + P25) or grey (TiO_2_ + MnO_2_ NP) color. They were cooled to room temperature and vigorously shaken in order to homogeneously disperse the P25 and MnO_2_ nanoparticles that had settled at the bottom.

Pure zinc oxide ZnO sol was synthesized according to Znaidi et al. [35,36]. Zinc precursor Zn(CH_3_COO)_2_∙2H_2_O as well as monoethylamine (molar ratio Zn(CH_3_COO)_2_∙2H_2_O: MEA of 1) were added to absolute ethanol (molar ratio a-EtOH: MEA of 58) and the mixture was stirred for 1 h at 70 °C. The obtained sol was perfectly transparent and could be used being cooled to room temperature. Its great stability allowed the sol to be kept for months.

#### 2.1.3. Preparation of Thin Films

Especially for sols synthesized by an organic method, alkali-free glass slides (AF32ECO, 75.0 mm × 25.0 mm × 0.7 mm, SCHOTT AG, Mainz, Germany) were chosen to obtain crystallization [19,20]. Identical slides were used for the aqueous sols to keep the substrate constant. To increase adherence, a surface treatment called “mild piranha treatment” or “piranha etching” was performed on the slides before dip-coating. Slides were first immerged in sulfuric acid and the system was cooled down to 0 °C. Then, hydrogen peroxide was carefully added (volume ratio H_2_SO_4_: H_2_O_2_ = 3) and the mixture was left for 12 h. The reaction led to the formation of H_2_SO_5_ or Caro’s acid, which is one of the strongest oxidants known. The slides were rinsed excessively in milli-Q water and dried under a stream of air. Because piranha treatment makes the surface of the slides far more hydrophilic, this treatment has not been used to clean slides prior to deposition of the organic sols. Instead, the slides for this application were first washed with an alkaline soap, then rinsed with water and ethanol (acetone for ZnO synthesis) and finally dried under a stream of air. Regardless of the cleaning treatment, slides were used within a few minutes of cleaning.

Dip-coating was performed on a Bungard RDC 21-K dip-coater (Windeck, Germany), allowing excellent and reproducible control of the dipping speed. The speed into the solution was set to 200 mm/min, while the removal from the solution speed was set to 60 mm/min for organic sols and 10 mm/min for aqueous sols. Films were dried in an air oven heated at 100 °C for 12 h (aqueous sol-gel synthesis) or calcined in an air furnace heated at 500 °C for 1 h (organic sol-gel synthesis).

#### 2.1.4. Powder Preparation

Since the total amount of matter deposited on the glass slides was too small for ICP-AES characterization, the dopant concentration was determined from the corresponding powder, which was produced by drying the sol in which glass slides were dipped. Sols of the doped photocatalysts were gelified and dried by air convection for 12 h, then either dried at 100 °C for 3 days (aqueous sols) or calcined for 4 h at 500 °C (organic sols).

### 2.2. Film Characterization

The crystallographic properties of the thin films were investigated by Grazing Incidence X-Ray Diffraction (GIXRD). The diffractometer (Bruker D8, Bruker, Billerica, MA, USA) using Cu-Kα radiation was operated at 40 kV and 40 mA with an incident beam angle of 1°.

The sizes of the crystallites, d_XRD_, were estimated from the XRD measurements using the Scherrer equation:

(5) $$d_{\mathrm{XRD}}=0.9\frac{\lambda }{B\cos\left(\theta \right)}.$$

where B is the full-width at half-maximum after correction of the instrumental broadening, λ is the wavelength (0.154 nm) and θ is the Bragg angle (rad). This measurement allows a qualitative comparison of the crystallite size between samples.

The adhesion of the coating to the substrate was assessed by a scotch tape test which consisted of sticking a common commercial scotch tape to the film and abruptly removing it. The adhesion was considered sufficient if the removal of the scotch tape did not cause visible damage to the film (this could be assessed by looking at the interference patterns made apparent upon exposure to visible light).

The thicknesses of the films were measured by mechanical profilometry (Veeco, Dektak 8 Stylus Profiler, Bruker). Data from a 3-mm-long scan with a vertical resolution of 0.1 nm were extrapolated to the whole coating.

The semiconductor band gaps were determined by diffuse reflectance spectrometry measurements on powders obtained from sols used to make the corresponding thin films (cf. 0). A Varian Cary 500 spectrophotometer with a BaSO_4_-coated integrating sphere was used to perform the measurements over the range 250 to 800 nm incident wavelength.

The UV–Vis spectra recorded in diffuse reflectance mode were transformed using the Kubelka–Munk function:

(6) $$F\left(R_{\infty }\right)=\frac{{\left(1-R_{\infty }\right)}^{2}}{2R_{\infty }}$$

where *R*_∞_ is defined as *R*_∞_ = *R*_sample_/*R*_reference_ with *R*_reference_ being the diffuse reflectance measured for the BaSO_4_ reference. Spectra were normalized by intensity. The following equation was used to estimate the band gap of the films:

(7) $$F\left(R_{\infty }\right)h\nu =C{\left(h\nu -E_{g}\right)}^{r}$$

where C is a constant and *r* is another constant depending on the type of electronic transition taking place, h is the Planck constant (6.63 × 10^−34^ J·s) and *ν* is the light frequency (Hz). The direct and indirect optical band-gap values were calculated by taking *r* = 0.5 for the direct allowed transition and *r* = 2 for the indirect allowed transition. The actual electronic transition mechanism is still under debate for anatase TiO_2_. Thus, both possibilities were considered.

### 2.3. Powder Characterization 

The actual amount of dopant in TiO_2_ powders was evaluated by inductively coupled plasma–atomic emission spectroscopy (ICP-AES, Varian Inc., Palo Alto, CA, USA) on a Varian Liberty Series II device.

The powders were digested using an alkaline fusion method. Two grams of sodium peroxide (Na_2_O_2_) and 0.2 g of sodium potassium carbonate (NaKCO_3_) were used to lower the melting point of 0.2 g of doped titania during fusion. The resulting slurry was dissolved in 6 mL of HNO_3_ before entering the ICP-AES device.

### 2.4. Photocatalytic Degradation of Methylene Blue

The photocatalytic activity of each film was evaluated through a common degradation test of methylene blue (MB). Twenty milliliters of a solution of 5.10^−5^ mol/L of MB was poured in Petri dishes, which were then covered by a quartz lid transparent to UVc radiation. This lid was essential to prevent the MB solution from evaporating.

Glass slides coated with a certain type of photocatalyst, as prepared in Section 2.1.3, were placed in the Petri dishes for testing. Three dishes containing one glass slide each were placed on a stirring plate at 50 rpm and exposed to UVc radiation for 4 h. One dish contained the MB solution, but no glass slide (blank sample), and underwent identical treatment. A fifth dish contained a glass slide but was protected from radiation and light (black sample). It was also stirred at 50 rpm on a stirring plate.

The UVc radiation was produced by a Philips TUV F17T8 lamp tube (Philips, Amsterdan, The Netherlands) emitting quasi-monochromatic radiation of wavelength 254 nm (UVc radiation). The irradiance of the lamp was measured with a STS-UV detector (Ocean optics, Largo, FL, USA) from Oceanview where Petri dishes were placed and amounted to 21 W/m^2^.

Two samples of 1.5 mL of the solution in each Petri dish were taken for analysis at 30 min and 4 h respectively. The sampling volume control ensured that the volume decrease in the Petri dishes at 30 min had no influence over the remainder of the experiment.

The experiments were carried out at ambient temperature under constant ventilation.

The MB concentration was evaluated by using a Genesys 10S-UV-Vis spectrophotometer [37], details are in the Supplementary Materials.

### 2.5. Photocatalytic Degradation of Pharmaceutical Products

Fifteen pharmaceutical products (PP) were investigated: 17-α-ethinyl-estradiol, β-estradiol, azithromycin, clarithromycin, erythromycin, sulfamethoxazole, trimethoprim, diclofenac, ibuprofen, tramadol, furosemide, carbamazepine, alprazolam, lorazepam, and metformin. A stock solution containing all of these molecules was prepared with a target concentration of 10 µg/L for each molecule, somewhat exceeding concentrations found in wastewater effluents. Each batch of 250 mL of stock solution stored in a bottle underwent 30 min. of a 200 mg/h flow of O_3_. This sufficed to completely eliminate 10 out of the 15 molecules that were being investigated. However, five molecules were resistant to this treatment: lorazepam, tramadol, alprazolam, ibuprofen, and metformin.

The exact same degradation procedure that has been applied to methylene blue (cf. Section 2.4) was then used for the pharmaceuticals, but with two differences: (1) the temperature was set and maintained at 15 °C throughout the experiment, since pharmaceutical products can be degraded naturally at ambient temperature, and (2) the UVc light intensity was equal to 3.20 W/m^2^, as a result of the different geometry of the setup used to control the temperature. The illuminated area of the slides corresponds to one face only. Indeed, glass is not transparent to UVc and the other face stayed in the dark during the experiment.

The pharmaceutical product concentrations were evaluated by UHPLC-MS/MS technique, details are in the Supplementary Materials.

### 2.6. Toxicity Tests

Toxicity tests were carried out according to the ISO 6341 norm using *Daphnia magna*, a micro-crustacean species. The animals were raised from ephippia and reached a homogeneous size and age. They were subsequently exposed to a solution of 50 µg/L of each pharmaceutical product mentioned in Section 2.5. After each step of the degradation process described in Section 2.5 (i.e., ozone treatment, ozone + UV treatment, and ozone + UV + photocatalytic treatment), the toxicity of the solution was assessed in the following way:

The solution was diluted with water at 100%, 50%, 25%, 12.5%, 6.25%, and 0% with respect to its initial concentration. Twenty *Daphnia magna* specimens were placed in each diluted solution for 24 h. The percentage of specimens still moving at the end of the test was then plotted against the aforementioned relative concentrations. A regression line was calculated using the least squares method, so that the relative concentration at which 50% of the specimens survive could be estimated: This concentration was called EC50. The toxicity T was then calculated as follows:

(8) $$T=\frac{1}{\mathrm{EC}50}$$

The results for the toxicity of the solution treated by photocatalysis correspond to the TiO_2_ + Ag sample, whose performance was the best amongst the photocatalysts investigated.

## 3. Results and Discussion

### 3.1. Composition of Samples

The ICP-AES results are presented in Table 1; they were collected on powders. No significant loss of dopant occurs during the synthesis or calcination step, except in the case of the MnO_2_ doping. Indeed, some MnO_2_ powder did not dissolve in the solution during the synthesis step.

### 3.2. Coating Aspect and Layer Thicknesses

For each type of sample, at least three different coated glass slides were measured; three measurements were taken at different places at the edge of each glass slide’s coating. The global average and standard deviation of these results is indicated in Table 1.

As was detailed earlier, two main sol-gel methods were used: organic and aqueous, depending on the solvent used.

Organic sols had a light yellowish color and were slightly more viscous than their aqueous counterparts. The dipping process was tailored to obtain thicknesses above 50 nm, as observed by Yu et al. [38]. All samples were thicker than 50 nm, apart from the Zn^2+^-doped sample, despite it being successively dipped four times (Table 1). After visual inspection, it is suspected that the layers are actually thicker in the center of the slides, because of the different colors produced by interference of light at the edge and at the center of the slides. This could not be verified with profilometry, as the curvature of the glass becomes significant on lengths higher than a few millimeters.

The roughness of most samples was similar to that of the glass which they were supported on (1 nm). The value given for the thickness is lower than the absolute roughness for TiO_2_ + P25. It indicates that big particles of P25 are at the surface of a layer whose thickness is actually lower than what is indicated in Table 1. TiO_2_ + P25 and TiO_2_ + MnO_2_ were however much rougher. This is due to the fact that they were the only two powder-based syntheses. This induced a higher surface area at the surface of the film. This exterior surface area has a better efficiency compared to the interior surface, as movement of chemical species is not diffusion-limited on the exterior surface, and since the light intensity is at its maximum there.

However, as the internal porosity of the film is unknown, no proof of an overall higher surface area for these two samples can be given. The relative efficiencies of interior and exterior surface areas are also difficult to compare.

### 3.3. Crystallographic Phases Composition

**Figure 1:**
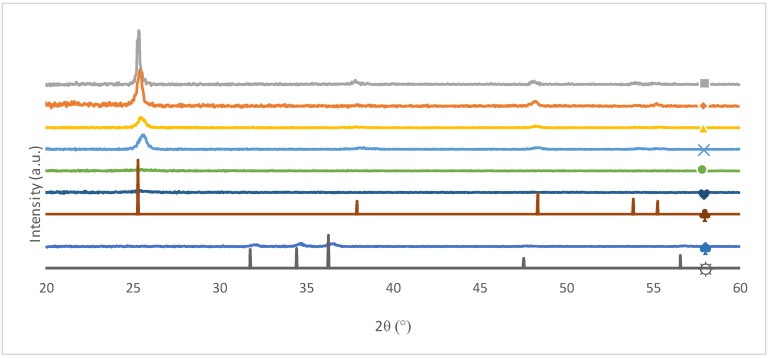
Grazing incidence diffractograms of all samples. ■ TiO_2_ org; ◆ TiO_2_ + Ag; ▲TiO_2_ + P25; ✕ TiO_2_ + MnO_2_; • TiO_2_ aq; ♥ TiO_2_ + Zn^2+^; ♣ Anatase (from database RRUFF R060277); ♠ ZnO; ☼ Zincite (from database RRUFF R050492).

Figure 1 represents the XRD patterns of all samples, and Table 1 shows the sizes of the TiO_2_ crystallites. Organic syntheses led to good crystallization of the TiO_2_ with large crystallites. For example, the Scherrer crystallite size for the TiO_2_ org sample was around 17 nm (Table 1), while aqueous syntheses (samples “TiO_2_ aq” and “TiO_2_ + Zn^2+^”) produced nanocrystallites more difficult to detect by XRD. Indeed, only small and highly broadened peaks are observed on, making it difficult to determine reliable crystallite sizes by the Scherrer formula. Nevertheless, TiO_2_ is not entirely amorphous in these samples, otherwise the sample would show only low catalytic degradation [38,39]. The anatase peaks present small displacements compared to the database standard. These displacements may come from the doping of the samples because the presence of a dopant distorts the TiO_2_ lattices.

Rietveld refinements could not be carried out because of the grazing incidence analysis used and the lack of a sufficient signal-to-noise ratio in the data. Anatase is the main phase in almost all coatings made by organic syntheses. This proves that the calcination step is suitable in these cases. Indeed, coatings produced by an organic route are calcined at 500 °C and this step promotes the formation of the anatase crystalline phase [40]. Concerning samples produced by an aqueous route (TiO_2_ aq. and TiO_2_ + Zn^2+^ samples), anatase remains the main crystalline phase as it is expected from the following synthesis [14]; the production of anatase at ambient temperature allows to reduce energy consumption because anatase crystallization is obtained without the need of a calcination step.

The TiO_2_ + P25 sample was modified by adding Evonik TiO_2_ P25 powder, which contains both anatase and rutile phases. However, the profilometry measurements for TiO_2_ + P25 show a significantly higher roughness than all other samples, meaning that the P25 powder is not incorporated in the anatase film. The resulting XRD signal is thus expected to be the weighted sum of the signals of the undoped TiO_2_ film and the pure P25. Because of the low amount of doping (10 wt%) and the low amount of rutile in P25 (15–20 wt%), the rutile phase was not detected in XRD diffractograms.

### 3.4. Diffuse Reflectance for Band-gap Measurement

Table 2 indicates the band gap energies calculated using diffuse reflectance. The values are consistent with what is found in the literature, both for anatase TiO_2_ [41] and ZnO [42]. While the exact band gap energies are not accurately determined, two considerations can be inferred.

Firstly, as all photocatalytic tests are performed under UVc radiation (254 nm), all catalysts are active in this range of energy. Secondly, the band gaps vary very little between all the samples. This is another indication that the aqueous syntheses led to anatase production, despite the anatase peak of the XRD spectra being barely visible for those samples (Figure 1).

### 3.5. Methylene Blue and Pharmaceutical Products Degradation

Table 3 summarizes the degradation percentages (d_X_) associated with each photocatalyst regarding the degradation of each pollutant after deducting the contribution of the photolysis caused by the sole exposure to UVc light and the adsorption (dark test), which was found to be negligible, i.e., the variation of the concentration of pollutants (MB or pharmaceutical products) was below 2%. The ranking of the performances differs strongly depending on the type of pollutant considered.

Among the 15 pharmaceutical products (PP) investigated, 10 are at least 90% degraded by the use of ozone (with or without UVc light, 254 nm) by 30 min. The UVc radiation used (254 nm) does not have sufficient energy to create ozone; hence, these two effects are independent. Only five PP require the addition of a photocatalyst: lorazepam, tramadol, alprazolam, ibuprofen, and metformin. Photocatalysis has thus been focused on these five compounds of interest.

Alprazolam was found to be the compound that resisted degradation the most (Table 3). Only TiO_2_ + Ag significantly degrades it: under the conditions described in Section 2.5, 40% of degradation was measured after 4 h, instead of 13% in the case of the second-best catalyst, TiO_2_ + P25. All other photocatalysts score between 0 and 10%. The Ag doping leads to the second highest average pharmaceutical products degradation.

The best performing samples are not the same for PPs as in the case of methylene blue degradation, where TiO_2_ + P25 has the highest degradation, followed by TiO_2_ + MnO_2_ and TiO_2_ aq. It must be noted that the various photocatalysts will be compared by their overall performance with little regard to the underlying mechanisms of photocatalysis. These mechanisms vary strongly among the various photocatalysts studied in this work.

The difference of performance ranking between the two tests hints at different mechanisms of degradation. Wu et al. [43] reviewed the mechanism of methylene blue degradation by TiO_2_. These authors suggest that water is adsorbed on the surface alongside the dye, which subsequently turns into •OH and •O_2_^−^ radicals and finally reacts with the dye. Contrarily, An et al. [44], established the kinetics of degradation of three other pharmaceutical products (norfloxacin, levofloxacin and lomefloxacin) as a series of rate constants linked mainly to free radicals •OH relegating adsorption/desorption to a secondary process. The first mechanism (degradation starting with adsorption) is enhanced by a higher affinity for the pollutant with the catalyst surface and by a higher surface area. This is indeed the tendency observed when comparing the values of roughness to the degradation efficiencies: While they seem to follow the same order in the case of methylene blue degradation (TiO_2_ + P25 > TiO_2_ + MnO_2_ > others), it is not the case for the degradation of pharmaceuticals. It is proposed that the adsorption-based mechanism is more preponderant for methylene blue degradation than pharmaceutical products degradation. The second mechanism (degradation caused by free radicals) requires a low recombination rate of e^−^/h^+^ pairs. It is precisely what doping TiO_2_ with Ag causes, as has been shown in other works [45].

TiO_2_ + Ag is in fact the only catalyst whose PPs degradation is higher for pharmaceutical product degradation than for methylene blue degradation. Some other samples such as TiO_2_ + MnO_2_, which are promising regarding the methylene blue degradation test, decrease sharply in the performance of degradation of pharmaceuticals.

### 3.6. Toxicity Tests

The toxicity tests are reported in Table 4. It is apparent that the toxicity decreases only after the photocatalysis treatment, despite the ozone treatment degrading most of the pharmaceuticals. However, it stays low from the beginning to the end of the process. One can infer that photocatalysis has a small positive impact on the acute toxicity of water. The impact could be bigger in the presence of more toxic molecules, at higher pollutant concentrations, or for longer durations for the photocatalysis step.

## 4. Conclusions

Different sol-gel syntheses were tailored to produce various metal oxide photocatalysts. Aqueous and organic syntheses were compared: While they both produced similarly thick layers, the latter performed better at degradation tests, whereas the former had the advantage of a cheaper, less energy-consuming production method. Indeed, compared to organic synthesis, the aqueous synthesis method does not require a calcination step for anatase crystallization.

Dopants were incorporated inside the metal oxides’ structures with relatively good efficiency, and the effect of dopants could be investigated. The most promising sample was Ag-doped TiO_2_, which had a similar performance compared to P25-doped TiO_2_ in pharmaceutical products degradation, but not in methylene blue degradation. Photocatalysis in advanced oxidation processes has been proven to be suitable for the degradation of some of the pharmaceutical products that could not be degraded by O_3_ and UVc light alone.

This work aims to be a basis for further developments for other similar syntheses to find more efficient compositions. The study also showed that the technology could, in principle, be implemented at a larger scale for wastewater treatments, if one proves that the coatings are durable.

## Figures and Tables

**Table 1:** Characterization of the coating by ICP-AES, profilometry, and X-ray diffraction.

Sample	Theoretical Dopant Content (wt%)	Actual Dopant Content (wt%)	Deposited Layers	Coating Thickness (nm) ^a^	Absolute Roughness (nm) ^a^	Scherrer Crystallite Size (nm)
TiO_2_ org (a)	- ^b^	- ^b^	2	57 ± 15	0.8 ± 0.3	17
TiO_2_ + Ag	1	0.92	2	66 ± 3	2.0 ± 0.2	109
TiO_2_ + P25 (a)	- ^b^	- ^b^	3	97 ± 25	155 ± 33	18
TiO_2_ + MnO_2_	5.42	3.49	2	89 ± 23	29.4 ± 8.4	18
ZnO (b)	- ^b^	- ^b^	2	56 ± 14	2.6 ± 0.4	80
TiO_2_ aq (a)	- ^b^	- ^b^	2	57 ± 10	1.5 ± 1.0	- ^c^
TiO_2_ + Zn^2+^	0.41	0.47	4	20 ± 4	0.7 ± 0.4	- ^c^

- ^a^: indicated with the standard deviation of three measurements; - ^b^: irrelevant; - ^c^: signal/noise ratio too low to measure.

**Table 2:** Band gap energies calculated by the Kubelka–Munk method from diffuse reflectance measurements on powders for the different allowed electronic transitions.

Sample	Direct Band Gap Energy (eV)	Indirect Band Gap Energy (eV)
TiO_2_ org	3.14	2.95
TiO_2_ + Ag	3.14	2.82
TiO_2_ + P25	3.13	2.93
TiO_2_ + MnO_2_	- ^a^	- ^a^
ZnO	- ^b^	3.04
TiO_2_ aq	3.24	2.87
TiO_2_ + Zn^2+^	3.29	2.91

- ^a^: powder too dark to be measured; - ^b^: not applicable.

**Table 3:** Contributions of the different catalysts on the degradation of methylene blue and different pharmaceutical products after 4 h, minus the effects of UVc only degradation and adsorption.

Sample	d_MB_ (%)	d_lorazepam_ (%)	d_tramadol_ (%)	d_alprazolam_ (%)	d_ibuprofen_ (%)	d_metformin_ (%)	d_PP_^a^ (%)
TiO_2_ org	24.4	33.4	13.7	6.7	29.7	21.9	21.1
TiO_2_ + Ag	29.1	30.4	13.7	40.1	11.9	51.1	29.4
TiO_2_ + P25	58.5	34.7	19.2	13.1	49.5	50.0	33.3
TiO_2_ + MnO_2_	51.7	30.8	17.0	5.7	27.4	27.0	21.6
ZnO	36.5	7.0	14.2	<1	19.8	<1	8.2
TiO_2_ aq	37.1	31.4	4.0	<1	14.1	<1	9.9
TiO_2_ + Zn^2+^	13.7	15.9	<1	<1	<1	14.0	6.0

^a^: grouping all the pharmaceutical products (PP) as one single type of chemical.

**Table 4:** Toxicity of a solution of the 15 pharmaceutical products concentrated at 50 µg/L after the different treatment steps.

Solution	Untreated	Treated with O_3_	Treated with O_3_ + UV	Treated with O_3_ + UV + Photocatalysis
Toxicity	1.32	1.33	1.33	1.19

## Supplementary Materials

The following are available online at http://www.mdpi.com/2079-4991/9/1/126/s1, Figure S1: Normalized Kubelka–Munk function F(R∞) with wavelength (λ) for the TiO_2_ org. sample, Figure S2: Evolution of (F(R∞)hν)2 as a function of the photon energy hν for the TiO_2_ org sample. The intersection of the linear part of the curve with the x-axis gives the band-gap value, Figure S3: Evolution of (F(R∞)hν)1/2 as a function of the photon energy hν for the TiO_2_ org sample. The intersection of the linear part of the curve with the x-axis gives the band-gap value.
