# Supplementary material for: Sol-gel Syntheses of Photocatalysts for the Removal of Pharmaceutical Products in Water
Source: Nanomaterials (Basel). 2019 Jan 20;9(1):126. doi: 10.3390/nano9010126 (PMC6358872; doi:10.3390/nano9010126)
Supplement: Supplementary file 1 [file nanomaterials-09-00126-s001.pdf]

Sol-gel Syntheses of Photocatalysts for the Removal of Pharmaceutical Products in Water

Artium Belet [1], Cédric Wolfs [1], Julien G. Mahy [1,*], Dirk Poelman [2], Christelle Vreuls [3], Nathalie Gillard [4] and Stéphanie D. Lambert [1]

<u>Supplementary materials:</u>

1. Band gap calculation

The sample optical properties are evaluated using diffuse reflectance spectroscopy measurements in the region of 300–800 nm with a Varian Cary 500 UV–Vis-NIR spectrophotometer, equipped with an integrating sphere (Varian External DRA-2500) and using $BaSO_4$ as reference. The UV–Vis spectra recorded in diffuse reflectance (R_{SAMPLE}) mode are transformed by using the Kubelka–Munk function (Eq. 1):

$$F(R_\infty) = \frac{(1 - R_\infty)^2}{2R_\infty} \quad (1)$$

where R_∞ is defined as $R_\infty = R_{SAMPLE}/R_{REF}$, with R_{REF} the diffuse reflectance measured for the $BaSO_4$ reference. To compare to each other, all spectra are normalized to 1.0 by dividing each spectrum by its maximum intensity. Using the well-known equation (Eq. 2):

$$F(R_\infty)h\nu = \left(C(h\nu - E_g)\right)^r \quad (2)$$

where C and r are constants, which depend on the optical transition mode. The direct and indirect optical band-gap values $E_{G\ DIRECT}$ (eV) and $E_{G\ INDIRECT}$ (eV) are obtained by plotting, respectively, $(F(R_\infty)h\nu)^2$ and $(F(R_\infty)h\nu)^{1/2}$ as a function of the photon energy $h\nu$ and by determining the intersection of the linear part of the curve and the x-axis. Because the actual electronic transition mechanism is still under debate for anatase TiO_2 (allowed direct and allowed indirect), both possibilities were considered.

An example for TiO_2 org sample is presented in Figures 1, 2 and 3. The evolution of the normalized Kubelka–Munk function $F(R_\infty)$ with wavelength (λ), is presented for TiO_2 org sample in Figure 1, the evolutions of $(F(R_\infty)h\nu)^2$ and $(F(R_\infty)h\nu)^{1/2}$ as a function of the photon energy $h\nu$ are plotted in Figure 2 and 3. The permitted direct and indirect band gaps are estimated by the intersection of the linear part of the curve and the x-axis.

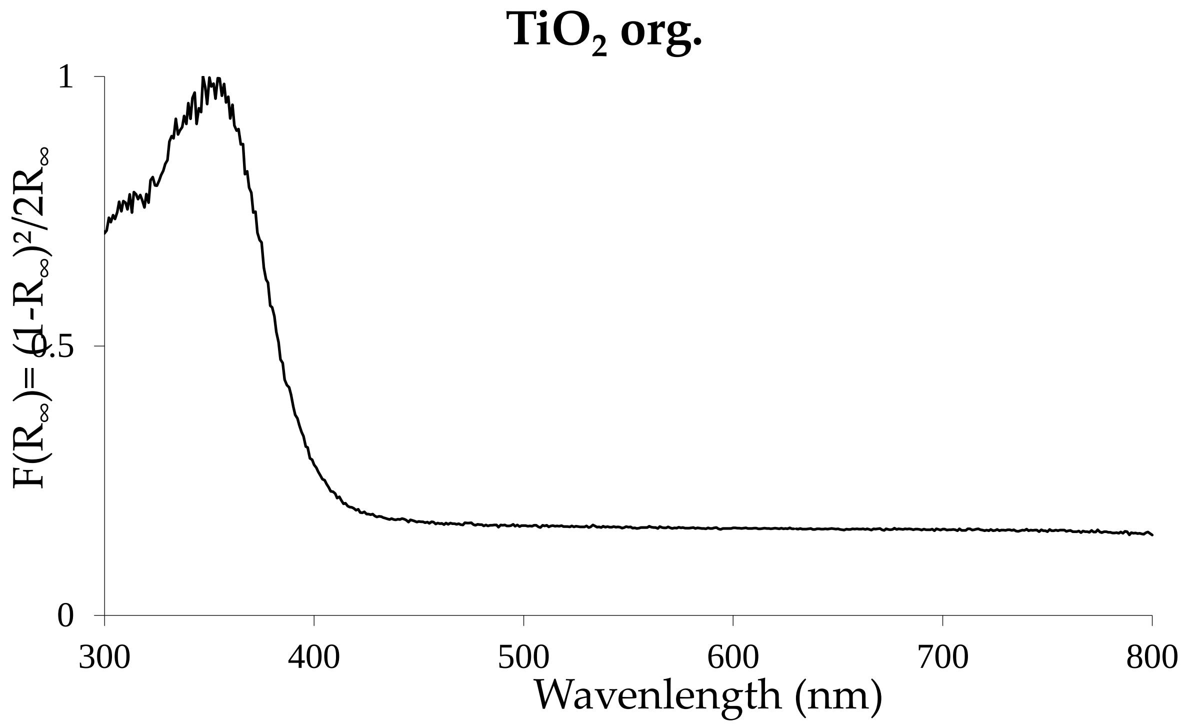

Figure 1. Normalized Kubelka–Munk function F(R∞) with wavelength (λ) for the TiO_2 org. sample.

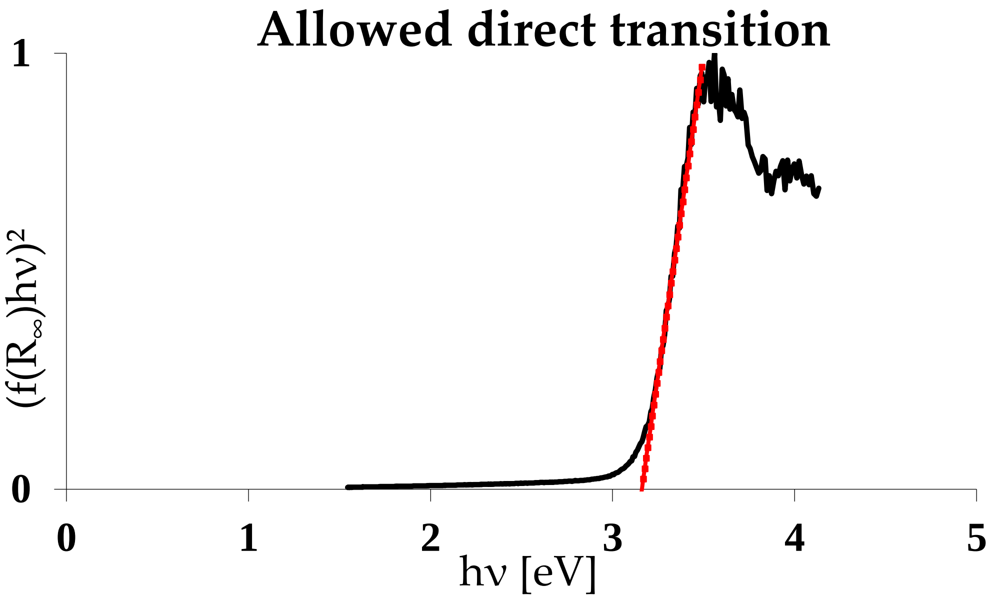

Figure 2. Evolution of (F(*R*∞)h*ν*)^2 as a function of the photon energy h*ν* for the TiO_2 org sample. The intersection of the linear part of the curve with the x-axis gives the band-gap value.

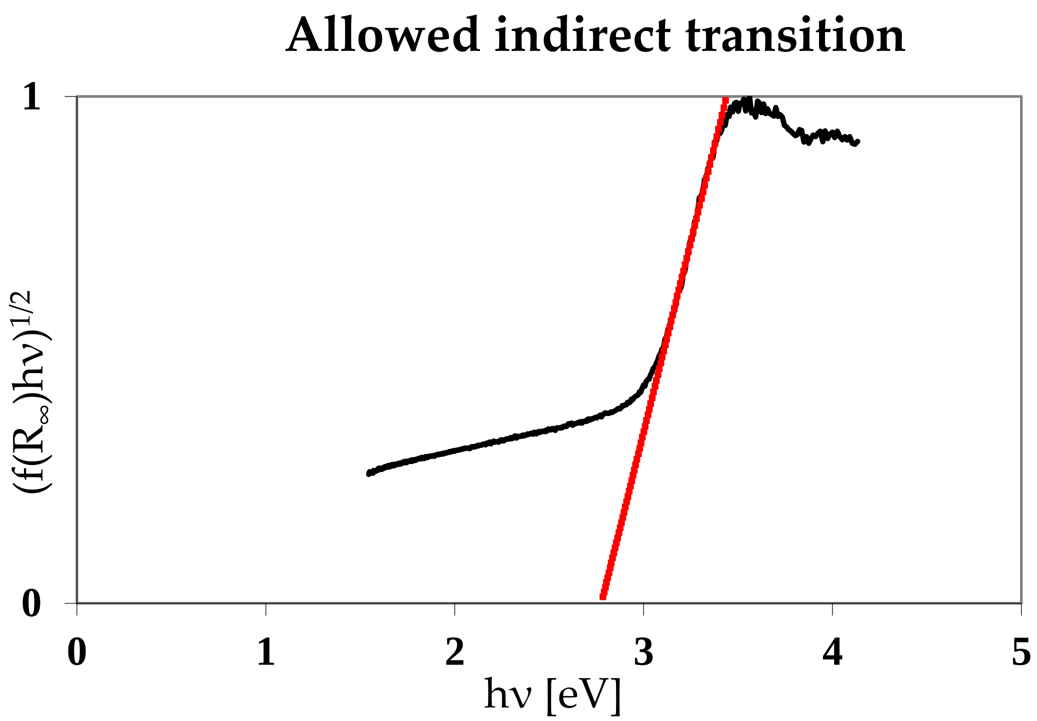

Figure 3. Evolution of (F(R∞)hν)1/2 as a function of the photon energy hν for the TiO2 org sample. The intersection of the linear part of the curve with the x-axis gives the band-gap value.

2. Detection method for photocatalytic tests

2.1. Methylene blue: UV-Vis spectrometry

The MB concentration was evaluated by the Beer-Lambert law [37] using a Genesys 10S-UV-Vis spectrophotometer, after calibration over the range of interest. The results are reported as percentages of degradation of MB, compared to the initial concentration of 5.10^{-5} mol/L, and were deduced from the absorbance of each sample solution.

2.2. Pharmaceutical products: UHPLC-MS/MS

Reagents and chemicals

The following standards were purchased from Sigma (St. Louis, MO, USA): alprazolam, carbamazepine, carbamazepine 10,11-epoxide, carbamazepine-d8, diclofenac, erythromycin, erythromycin-d6, ethinyloestradiol, 17-β-oestradiol, furosemide, ibuprofen, ibuprofen-d3, sulfamethoxazole, tramadol, trimethoprim, metformin, metformin-d5, lorazepam, lorazepam-d3, clarithromycin, clarithromycin-d3, azithromycin, azithromycin-d3, sulfamethoxazole-d4, tramadol-d6, furosemide-d5 were purchased from TRC Canada (Toronto, Canada). Trimethoprim-d9 and diclofenac-C13 were obtained from Witega (Berlin, Germany) while 17-β-oestradiol-d4 was purchased from RIKILT (Wageningen, Netherland).

Extraction protocol

For concentrations of pharmaceuticals in our range of interest (around 10 µg/L), no solid phase extraction was needed. The samples were directly placed in HPLC vials.

The quantification method was based on matrix-matched calibration curves with 11 concentration levels (0, 10, 25, 50, 100, 500, 1000, 2500, 5000, 7500, 10000 ng/L). A regression model was applied to the calibration data set. With each extraction batch, a quality control (QC) sample was also analyzed.

Sample analysis by UHPLC-MS/MS

The UHPLC equipment consisted of a Waters Acquity system (Waters, Milford, USA). Chromatography was performed on an Acquity UPLC® HSS C18 1.8 µm column (150x2.1 mm, Waters, Milford, USA). During UHPLC analysis, the column was maintained at 50 °C and the samples at 10 °C. For each sample, two injections were performed on the same column, one for negative (injection volume 35 µL) and one for positive (injection volume 20 µL) electrospray analysis, with an optimized mobile phase for each injection. In both cases, the flow rate was 0.5 mL/min. For positive electrospray ionization (ESI), mobile phases A and B were, respectively, 0.1% formic acid in water and 0.1% formic acid in acetonitrile. The applied elution program was a two-linear-step gradient: 0–5 min, linear gradient from 90 to 0% A; 5–5,5 min, linear gradient from 0 to 90% A; 5,5–7 min, from 90% to 0% A; and finally reconditioning of the column until 7 min.

For negative ESI, mobile phases A and B were, water and acetonitrile, respectively. The applied elution program was a linear gradient: 0–4 min, from 75% to 0% A; 4–4.5 min, linear gradient from 0 to 75% A; and finally reconditioning of the column until 6 min.

The MS equipment consisted of a XEVO TQS-micro (Waters, Milford, USA). The analysis was performed either in positive or negative ion electrospray mode. Multiple reaction monitoring was carried out. Two transitions were followed for most PPs, the first being the quantifier and the second the qualifier. Nitrogen was used as the cone gas and the desolvation gas at flow rates of 250 and 1200 L/h, respectively. The other MS–MS parameters were: capillary voltage, 2 kV; source temperature, 150 °C; desolvation temperature, 500 °C; collision gas pressure, 3.10^{-3} mbar. Multiple reaction monitoring was carried out. Data collection and subsequent processing were performed with TARGETLYNX software (Waters, Milford, USA).
